# Imaging and positioning through scattering media with double-helix point spread function engineering

**DOI:** 10.1117/1.JBO.28.4.046008

**Published:** 2023-04-25

**Authors:** Jingjing Gao, Pengwei Wang, Wenwen Li, Xuyu Zhang, Chunyuan Song, Zhentao Liu, Shensheng Han, Honglin Liu

**Affiliations:** a Chinese Academy of Sciences, Shanghai Institute of Optics and Fine Mechanics, Key Laboratory of Quantum Optics, Shanghai, China; b University of Chinese Academy of Sciences, Center of Materials Science and Optoelectronics Engineering, Beijing, China; c University of Science and Technology of China, Hefei National Laboratory, Hefei, China; d University of Chinese Academy of Sciences, Hangzhou Institute for Advanced Study, Hangzhou, China

**Keywords:** scattering medium, double-helix point spread function, penetration thickness, three-dimensional localization, deconvolution

## Abstract

**Significance:**

Double-helix point spread function (DH-PSF) microscopy has been developed for three-dimensional (3D) localization and imaging at super-resolution but usually in environments with no or weak scattering. To date, super-resolution imaging through turbid media has not been reported.

**Aim:**

We aim to explore the potential of DH-PSF microscopy in the imaging and localization of targets in scattering environments for improved 3D localization accuracy and imaging quality.

**Approach:**

The conventional DH-PSF method was modified to accommodate the scanning strategy combined with a deconvolution algorithm. The localization of a fluorescent microsphere is determined by the center of the corresponding double spot, and the image is reconstructed from the scanned data by deconvoluting the DH-PSF.

**Results:**

The resolution, i.e., the localization accuracy, was calibrated to 13 nm in the transverse plane and 51 nm in the axial direction. Penetration thickness could reach an optical thickness (OT) of 5. Proof-of-concept imaging and the 3D localization of fluorescent microspheres through an eggshell membrane and an inner epidermal membrane of an onion are presented to demonstrate the super-resolution and optical sectioning capabilities.

**Conclusions:**

Modified DH-PSF microscopy can image and localize targets buried in scattering media using super-resolution. Combining fluorescent dyes, nanoparticles, and quantum dots, among other fluorescent probes, the proposed method may provide a simple solution for visualizing deeper and clearer in/through scattering media, making *in situ* super-resolution microscopy possible for various demanding applications.

## 1 Introduction

Optical imaging has gained widespread use in biomedical imaging because of its high resolution and capacity to provide diverse information. However, the propagation of light in biological tissues is diffused by scattering, resulting in limited penetration depth and poor resolution. Achieving super-resolution imaging through/in scattering media is highly desirable but extremely challenging. Various fluorescence microscopies have achieved resolutions down to nanometers using sparsely excited fluorescent particles and their corresponding algorithms. However, such techniques are limited to discrete cells *in vitro* rather than *in vivo*. Understanding the functions and mechanisms of cells in their microenvironment is critical. Therefore, there is an urgent need for *in-situ* super-resolution imaging.

Several techniques have been proposed to overcome the challenges posed by scattering. Wavefront shaping^1^^–^^3^ and transmission matrix measurement^4^^–^^6^ can compensate for or characterize scattering; however, they require either a navigation star or access to the other side of a stationary scattering medium. The deconvolution imaging technique^7^^,^^8^ requires prior measurements of the point spread function (PSF) of the scattering medium; therefore, it is not ideal for dynamic biological tissues. Speckle autocorrelation imaging^9^^–^^11^ is immune to the motions of scattering media, but it has a limited field of view determined by the optical memory effect range.^12^^–^^14^ These methods and techniques are unsuitable for super-resolution microscopy through or in biological tissues, particularly noninvasively.

Microscopic imaging techniques have been developed to enhance the resolution and penetration depth. Confocal microscopy^15^^–^^17^ is primarily used for imaging live cells cultured *in vitro* and superficial areas of fixed tissues owing to its point scan and limited penetration capabilities. Structured light illumination microscopy,^18^^–^^20^ which can achieve a resolution of half the diffraction limit by illuminating the sample with a modulated streak structure light, requires precise modulation and complex reconstruction algorithms and does not significantly improve the penetration depth. The penetration depth is greater for longer wavelengths, which leads to the development of two/multiphoton microscopies.^21^^–^^24^ The penetration depth can reach ∼500  μm for 1300 nm photons,^25^ with an equivalent optical thickness (OT) of 2. Despite these improvements, the penetration depth and resolution are still limited to observing subcellular structures and molecular arrangements in cells beneath or in scattered biological tissues.

After introducing the double-helix point spread function (DH-PSF),^26^ Pavani et al. optimized the method and proposed DH-PSF microscopy to enable three-dimensional (3D) fluorescence imaging in super-resolution.^27^^,^^28^ Because of its excellent axial positioning ability, DH-PSF microscopy has been widely applied in in-depth estimation,^29^^,^^30^ particle tracing,^31^^–^^33^ and other applications.^34^^–^^36^ However, most DH-PSF microscopy has been conducted in weak or nonscattering environments, and its performance in strong scattering environments has not yet been explored.

We developed DH-PSF microscopy to image through biological tissue layers by taking advantage of the easier recognition of the double-spot structure rather than a single spot in the background of speckles. Through simulations in a scattering environment, we demonstrated excellent localization capability within an OT of 5. We then built an experimental system and achieved a transverse (x-y) localization accuracy of 13 nm and an axial localization accuracy of 51 nm. Utilizing a specially designed algorithm, we successfully reconstructed 3D images of multiple fluorescent microspheres through an eggshell membrane and a piece of an onion epidermal tissue layer, with precise localization of all microspheres and improved visualization of membrane structures owing to axial filtering in deconvolution. Our approach may provide a solution for *in situ* and *in vivo* 3D super-resolution imaging of biological tissues.

## 2 Methods

### 2.1 Generation and Optimization of DH-PSF

Traditionally, the DH-PSF scheme is created by a linear superposition of LG modes with equal weights. An LG beam is a vortex beam with unique parameters, such as the vortex phase and spin angular momentum. Compared with a conventional Gaussian beam, a vortex beam has better resistance to turbulence interference during propagation.^37^^,^^38^ Accordingly, the DH-PSF maintains a relatively complete beam shape through the scattering medium. Under the modulation of the DH-PSF, a Gaussian spot on the image plane (i.e., the PSF of a conventional imaging system) is transformed into two discrete centrosymmetric spots. The size and shape of the double spot were preserved, and its azimuthal angle changed as the point target deviated axially from the initial object plane. The relationship between the azimuthal angle θ and axial distance z is^26^

$$\theta =2\;\arctan[\frac{z_{\mathrm{obj}}^{2}}{z_{0}}(\frac{1}{z}-\frac{1}{z_{\mathrm{obj}}^{\text{focus}}})],$$ (1)

where $z_{0}$ is the Rayleigh length, $z_{\mathrm{obj}}$ is the distance from the object plane, and $z_{\mathrm{obj}}^{\text{focus}}$ is the object plane distance from the entrance pupil.

The direct superposition of LG modes introduces both phase and amplitude modulations into the mask. However, most wavefront modulation devices are sensitive to either the amplitude or the phase, resulting in low energy efficiency. To enhance energy efficiency, the mask is usually optimized to be phase-only.^27^ Here, we adopted the optimization method described in Ref. 27, combined with the Gerchberg–Saxton (GS) algorithm, and obtained an energy efficiency enhancement of ∼30 times.

### 2.2 Evaluation of Localization Accuracy in a Scattering Environment

**Fig. 1:**
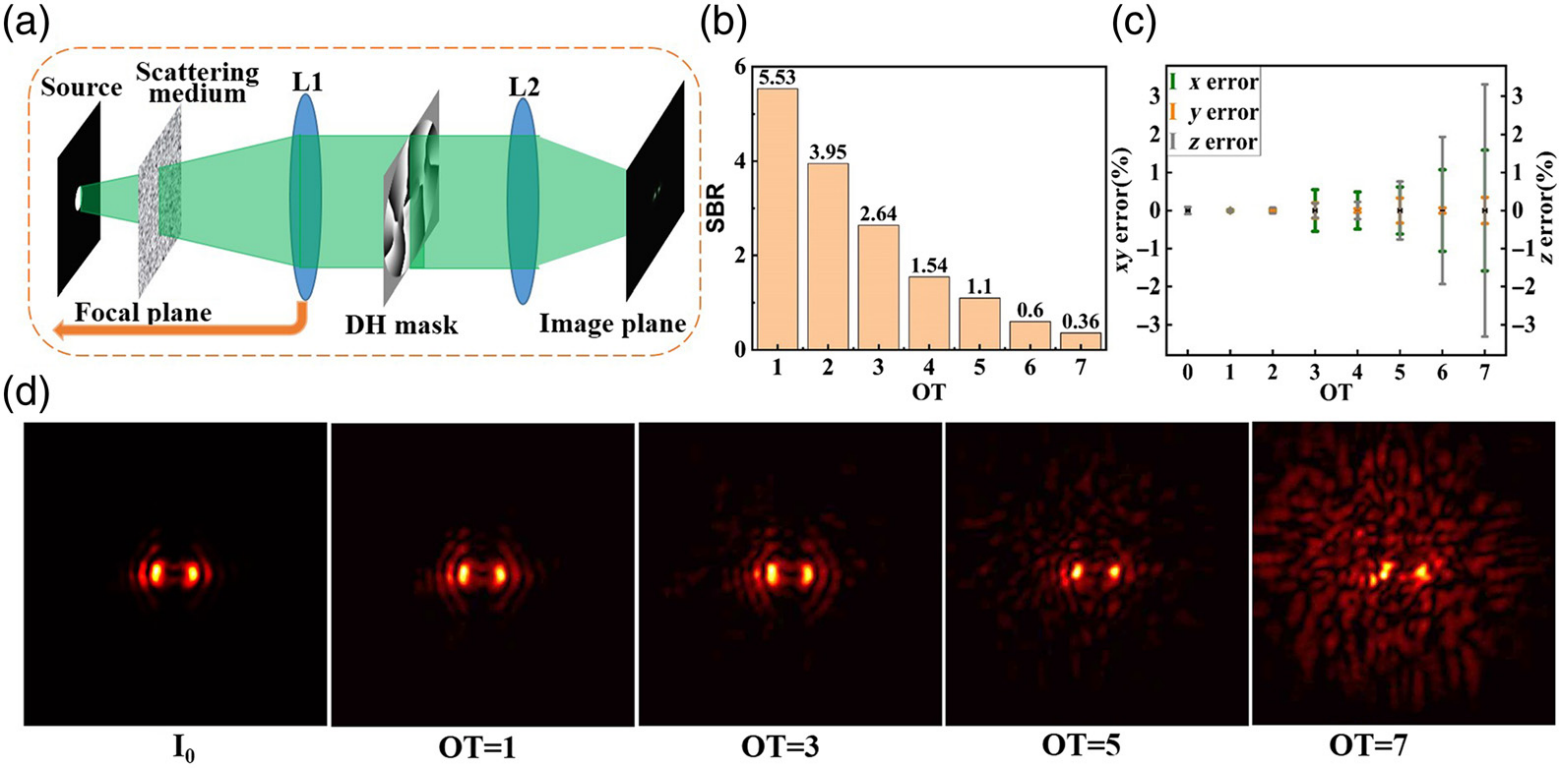
Simulation of DH-PSF modulation in a scattering environment. (a) Simulation configuration: a point source on the front focal plane of L1 illuminates a scattering medium and then enters into the 4f system with a DH-PSF mask inserted into the middle focal plane. The image plane is the back focal plane of L2. (b), (c) SBRs and localization errors at different OTs, respectively. (d) Recorded patterns on the image plane at different OTs.

In this study, we conducted simulations of DH-PSF modulation in a scattering environment and systematically investigated the effect of scattering on positioning accuracy. The scattering medium was simulated using a multilayer phase-mask model constrained by the spatial power spectral density^39^ with an anisotropy factor g=0.95. Different thicknesses and optical depths were considered, with all scattering effects concentrated on a plane and represented as a single random phase mask. The configuration of the simulation is shown in Fig. 1(a). The DH-PSF mask was placed on the middle plane of a 4f system composed of L1 and L2.

**Fig. 2:**
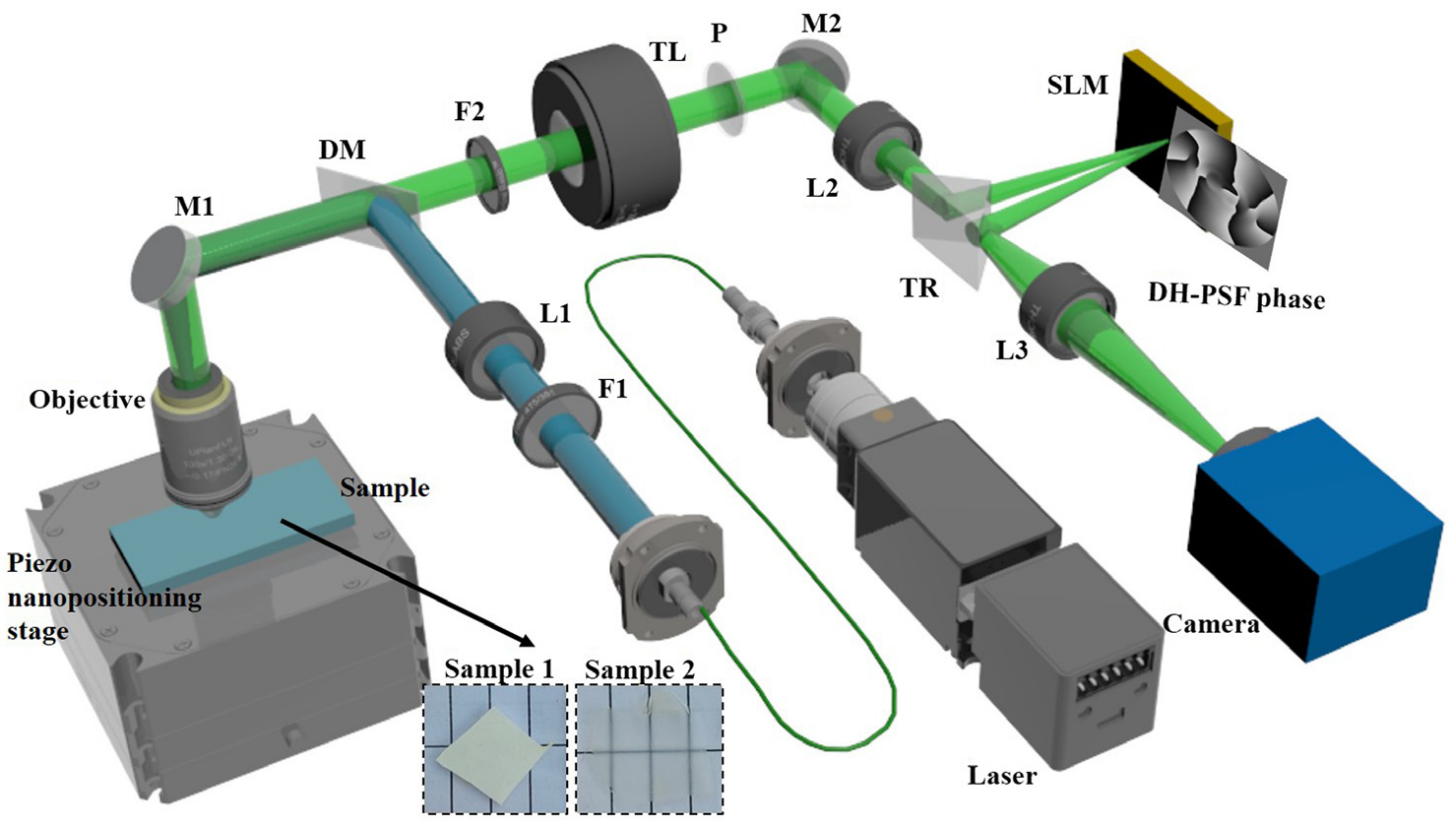
Experimental setup. A 488-nm laser beam is filtered and collimated and then coupled into an objective to illuminate the sample. The objective collects the fluorescent light and subsequently passes through the DM, TL, and the PSF engineering module before being collected by the camera. M1 – M2: mirrors, F1 – F2: filters, L1 – L3: lenses, TL: tube lens, TR: triangle reflector, DM: dichroic mirror (with a cut-on wavelength of $\lambda_{c}=500\;\mathrm{nm}$), P: polarizer, SLM: spatial light modulator. Two photographs of samples 1 and 2 placed on a grid pattern are inserted to show their visual turbidity.

The back focal plane of L2 is the image plane, and Fig. 1(d) shows the intensity patterns of the DH-PSFs of a point source at various OTs. When the OT is small, the double spot maintains its shape well; however, as the OT increases, the distortion of the double spot becomes more severe, resulting in more energy escaping from the two spots and contributing to random speckles. However, if the signal-to-background ratio (SBR)^40^ is greater than a certain value, the localization algorithm can still operate normally with high accuracy. Figure 1(b) shows the decay of the SBR with increased OT, and Fig. 1(c) shows the calculated localization error at different OTs. At OT=6, the SBR drops to ∼0.6, and the positioning errors in the x-, y-, and z-directions exceed 1%. An SBR>1 is typically required to ensure positioning accuracy, corresponding to an OT of ∼5 and a transmission rate of 0.7%. At OT=5, the localization errors in all axes are <1%; thus, the positioning accuracy is considered to be equivalent to that for nonscattering environments.

The simulation results prove that DH-PSF can be utilized for localization in scattering environments. However, in experiments, the physical thickness of the scattering medium cannot be ignored, and it is necessary to choose thin scattering layers considering the limited working distance of the microscope objectives.

## 3 Experiment

### 3.1 Experimental Setup

A sample of buried fluorescent microspheres ($E_{x}/E_{m}:488/550\;\mathrm{nm}$, GF1000C, Huge Biotechnology) was illuminated by a 488-nm excitation beam from a semiconductor laser (Lambda beam 488 – 200, RGB Laser Systems), as shown in Fig. 2. The emitted fluorescence light was collected using a 100× microscopic objective (numerical aperture: 1.3, RMS100X–PF, Olympus) paired with a tube lens with a focal length of f=200  nm. The exit image plane was relayed by a 4f system consisting of lenses L2 and L3 onto a camera (Edge 4.2, PCO). The optimized DH-PSF phase distribution was displayed on a reflective phase-only spatial light modulator (SLM) (LETO, Holoeyes), placed on the back focal plane of L2. A triangular reflector was applied to approximate the norm incidence to ensure the proper use of the SLM at nearly normal incidence. The sample was placed on a piezo nanopositioning stage (P18.XYZ200S, Core Morrow). To suppress the environmental background, bandpass filters F1 (LL01-488-25, Semrock) and F2 (BLP01-488R-25, Semrock), along with a dichroic mirror (Di03-R488-t1-25◊36, Semrock), were used.

Two different samples were prepared. One consisted of several fluorescent microspheres randomly scattered on a slide and covered by an eggshell membrane with a thickness of ∼20  μm. In contrast, the other consisted of several microspheres covered by a layer of onion epidermal tissue with a thickness of ∼100  μm. Unlike the eggshell membrane, the surface of the onion epidermal tissue was rougher, with a greater chance of microspheres attaching to the valleys, leading to axial depth variations. The thicknesses of both samples were smaller than the working distance of the objective lens. The SBRs of the modulated double-spots for both samples were >1, satisfying the condition for accurate positioning.

### 3.2 System Calibration

In DH-PSF imaging and positioning, the lateral position is determined from the midpoint of the line connecting the double spots, whereas the axial position is estimated from the azimuth angle of the line. Typically, the angle is mapped to an axial position to calibrate the system. To prepare the calibration sample, a solution of fluorescent microspheres with an average diameter of 1  μm was diluted 5000 times, and a drop of the diluted solution was placed on a microscope slide with a pipette. After evaporation, discrete fluorescent microspheres remained on the slide and were used as calibration samples. The samples were fixed on the piezo nanopositioning stage, and the default z=0 corresponded to the focal plane.

**Fig. 3:**
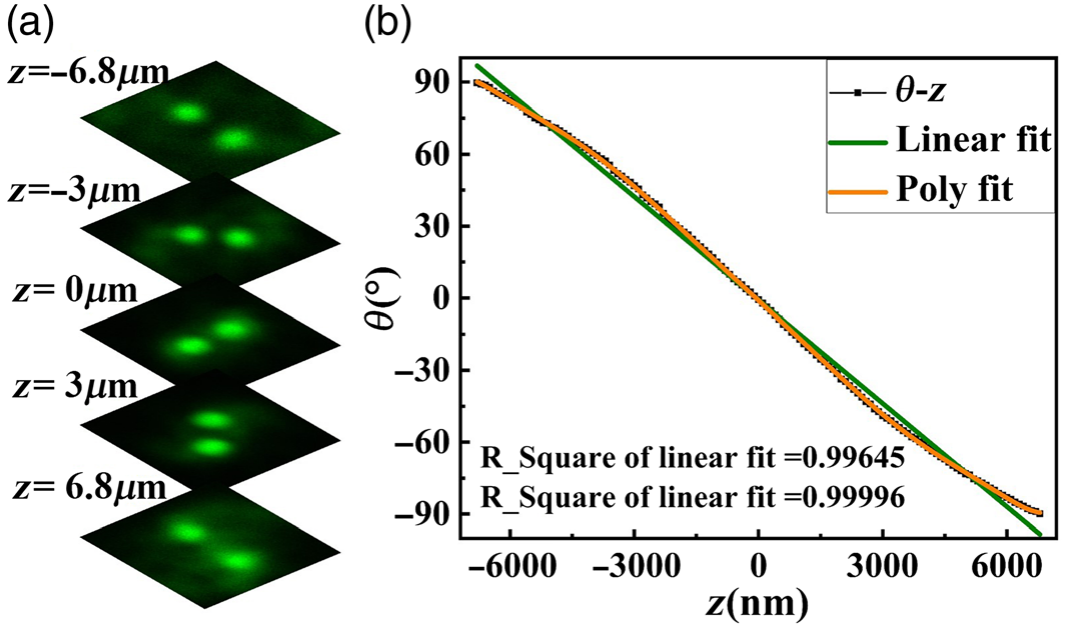
Calibration of the experimental system. (a) Modulation patterns of a selected microsphere at several z positions. (b) Calibration curves of angle θ with axial position z.

To obtain DH-PSF modulated patterns, the stage was scanned along the z axis from −6.8 to 6.8  μm with a step size of 100 nm, and 137 frames were recorded. Figure 3(a) shows the patterns of a selected microsphere corresponding to several z positions. As the z-value changes within the scanning range, the modulated double spot rotates around its center, and the rotating angle varies from $-\frac{\pi }{2}$ to $\frac{\pi }{2}$. More side lobes appear at a larger deviation from z=0. Figure 3(b) shows that θ is approximately proportional to z, and a linear fit of the θ−z relationship yields a coefficient of determination of R_Square = 0.99645. However, a polynomial fitting curve is a closer representation of the relationship, with a higher R_Square = 0.99996. A polynomial curve was adopted in our investigation to enhance the accuracy of the axial localization. In addition, the system was calibrated in advance for lateral drifts to ensure and estimate imaging and localization accuracy.

### 3.3 Quantification of Localizing Accuracy

**Fig. 4:**
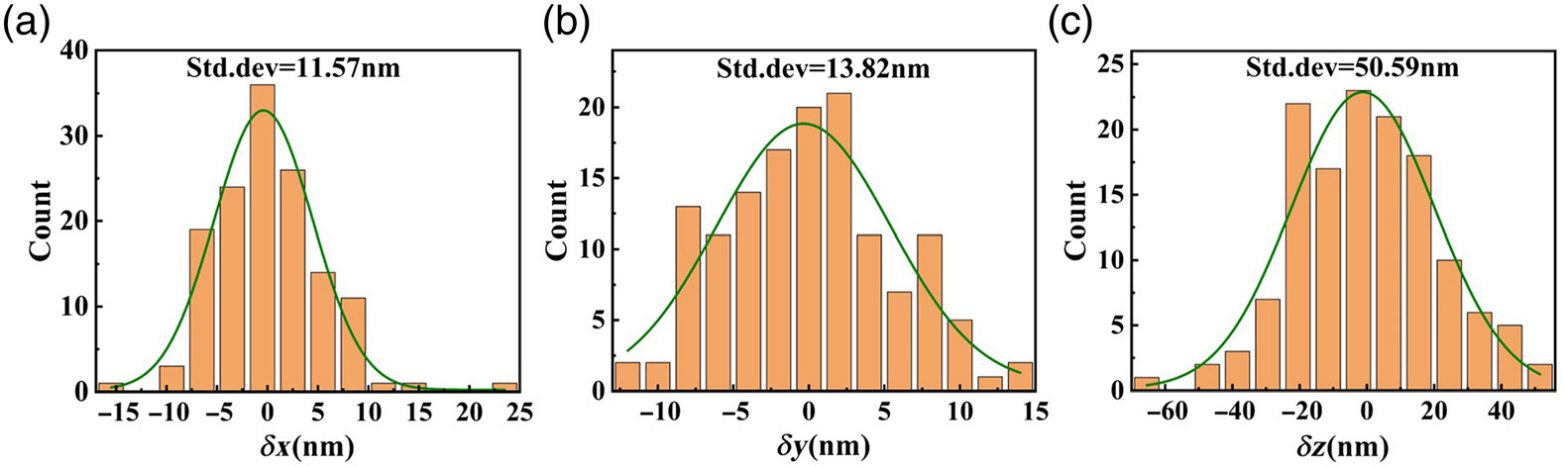
Localization accuracy in the x, y, and z directions. (a)–(c) Distributions of positioning in the x, y, and z directions and corresponding localization accuracy estimated from the fitted curve.

The recorded patterns of 137 positions were adopted for quantifying the localization accuracy, with a preset interval of 100 nm for stage scanning as the reference value. Statistical analysis was performed on the error values in the x-, y-, and z-directions, and the resulting histogram distribution diagrams are shown in Fig. 4. The full-width at half-maximum value of the Gaussian fitting curve represents the positioning accuracy, which is 13 nm in the x- and y-axes directions and 51 nm in the z axis direction.

### 3.4 Algorithms

Super-resolution images can be reconstructed by fusing hundreds or thousands of frames of sparsely excited fluorescent particles far smaller than the diffraction limit. However, in our study, the fluorescent microspheres were larger than the diffraction limit of λ/2 NA=211  nm, and the membranes in our samples had dense and continuous structures. In our experiment, we positioned the fluorescent microspheres by fitting a double Gaussian spot and used deconvolution based on DH-PSF to reconstruct the membrane structure around the microspheres. Because the focal plane of the microscopic objective lens was fixed in our experiment, we moved the sample layer-by-layer to the focal plane of the microscopic objective lens during the movement process. By choosing the in-focus DH-PSF to deconvolve the recorded data, a clear image of each layer is recovered, and a 3D image of the sample is obtained by stacking. The in-focus DH-PSF is a double-spot pattern at the plane of z=0. We also observed that deconvolution of the in-focus DH-PSF blurs out-of-focus sections and enhances in-focus sections, thereby improving image quality. Such convolution processing functions as optical sectioning.

## 4 Results

### 4.1 Imaging and Localization through Eggshell Membrane

**Fig. 5:**
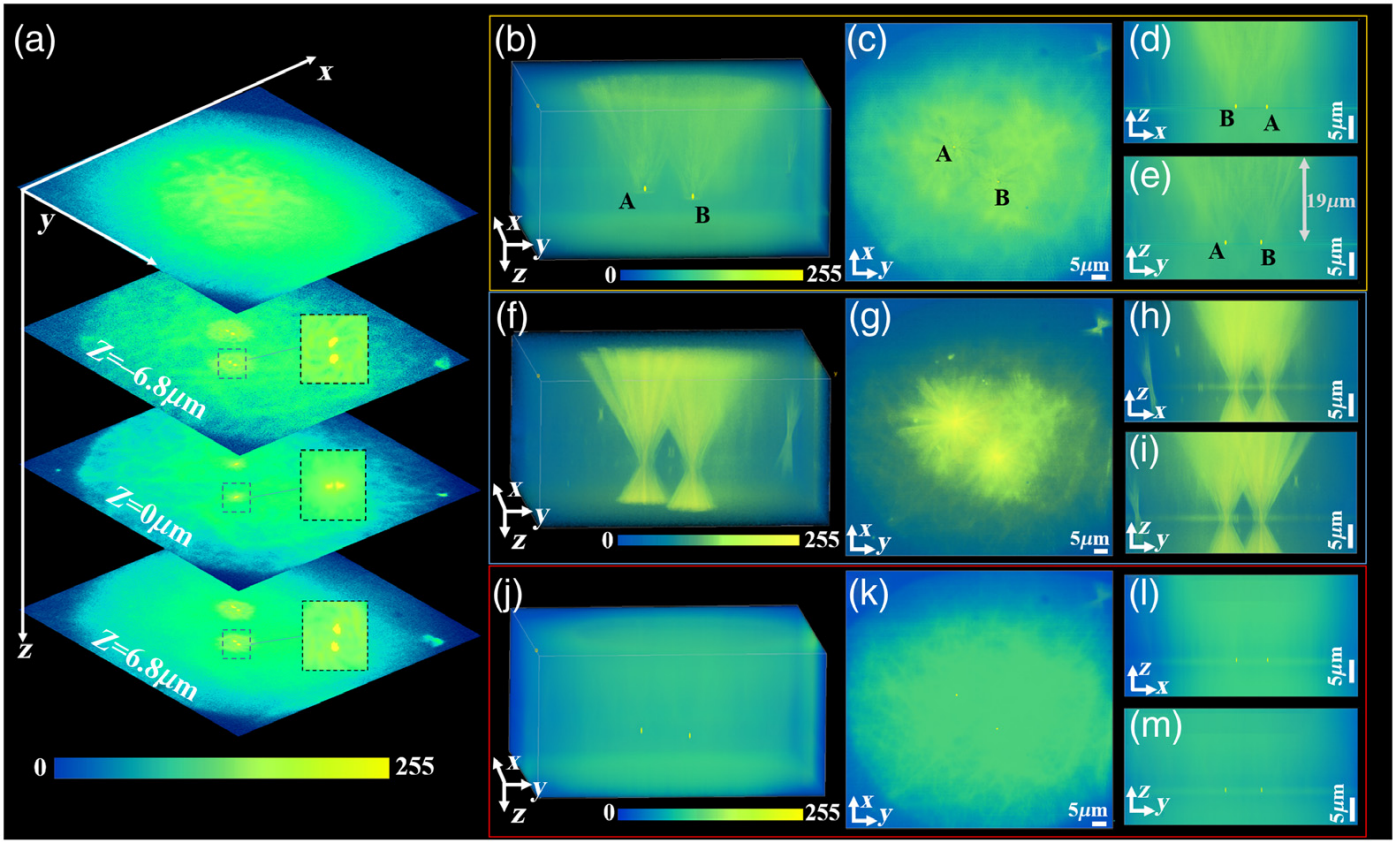
Imaging and localization through eggshell membrane. (a) Selected frames during scanning with the DH-PSF mask on the SLM. The double-spot rotates as the value of z changes, as seen in the zoom-ins. The color bar is shown at the bottom. (b) 3D image of sample 1 reconstructed from our modified DH-PSF method. (c)–(e) Projection views in the x-y, x-z, and y-z planes, respectively, with corresponding color and scale bars. (f)–(i) Results of conventional microscopy. (j)–(m) Results of the conventional DH-PSF technique. Compared with conventional microscopy, conventional DH-PSF accurately localizes discrete fluorescent particles but has worse image quality of the eggshell membrane with less distinguishable details. In contrast, our method differentiates the structures of protein fibers in the eggshell membrane (Video 1; Video 2).

Sample 1 was fixed on the nanopositioning stage, which was adjusted to change the object distance to obtain the finest double spot, and its initial position was set to z=0. The stage was scanned uniformly toward the objective lens from z=−19.6  μm to z=8.3  μm, with images recorded at z=−19.6  μm and subsequently at each 100 nm interval. The recorded images at different positions are presented in Fig. 7 (Video 1); the images of conventional microscopy, without the DH-PSF mask on the SLM, are provided in Fig. 8 (Video 2) for comparison. The four frames of images from Fig. 7 (Video 1) are shown in Fig. 5(a). The image at z=0 exhibits a sharp double-spot structure. As the absolute value of z increases, the azimuth angle of the double spot changes. The position of each fluorescent microsphere was calculated, and each frame was deconvoluted using the DH-PSF at z=0 and stacked together to obtain a 3D image, as shown in Fig. 5(b). The membrane was illuminated with fluorescent light from microspheres. Because of DH-PSF deconvolution’s excellent optical sectioning performance, the microspheres, their relative position to the membrane, and the membrane’s internal structures can be well differentiated. In contrast, the images obtained by conventional microscopy [Figs. 5(f)–5(i)] and conventional DH-PSF microscopy [Figs. 5(j)–5(m)] are blurred.

In the conventional DH-PSF scheme, sparsely distributed fluorescent particles, which are much smaller than the diffraction limit, can be localized with an accuracy of nanometers or tens of nanometers, and the final image is a fusion of numerous frames of discretely excited fluorescent particles, which are bound to targets of interest, organelles, and structures in cells. The resolution is determined by the localization accuracy, thus achieving super-resolution. However, in our case, the fluorescent microspheres were larger than the diffraction limit, and the eggshell membrane was not fluorescently stained. Images were obtained by deconvolution. During scanning, only the in-focus plane has a double-spot PSF of θ=0  deg, at z=0, whereas the out-of-focus planes (away from z=0) have rotated double-spots. After deconvoluting the double-spot PSF scheme at z=0, the image of the in-focus frame is enhanced, whereas the out-of-focus frames are blurred, thus achieving optical sectioning capability. The positions of the microspheres through the scattering layer are still localized, as in conventional DH-PSF engineering, with unprecedented accuracy in scattering environments. Figures 5(c)–5(e) show the projection views on the x-y, x-z, and y-z planes. The estimated thickness of the eggshell membrane from the y-z projection, as shown in Fig. 5(e), is ∼19  μm. Microspheres A and B are calculated to be 19.62 and 19.63  μm away from the top surface of the eggshell membrane, respectively.

### 4.2 Imaging and Localization through Onion Epidermis Tissue Layer

**Fig. 6:**
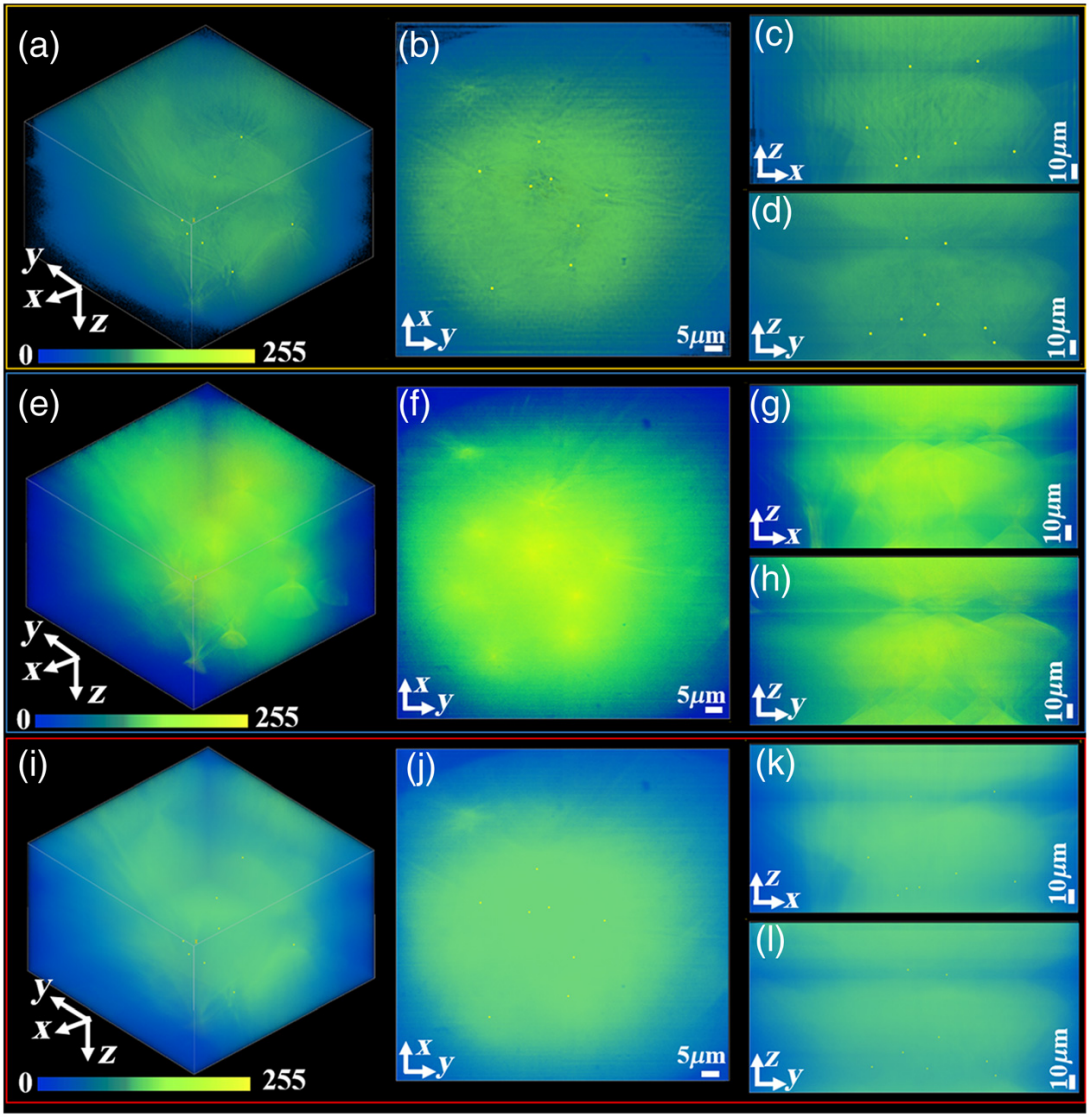
Imaging and localization of multiple microspheres through the onion epidermal tissue. (a) The reconstructed 3D images of multiple fluorescent microspheres in sample 2 by utilizing of our method. (b)–(d) Projection views in the x-y, x-z, and y-z planes, respectively, with corresponding scale bars. (e)–(h) Results of the conventional microscopy. (i)–(l) Results of the conventional DH-PSF technique. Our method provides the best image quality while preserving super-resolution localization capability (Video 3; Video 4).

For sample 2, a larger scanning interval of 500 nm was used because of its thicker structure. The scanning range was from z=−34.5  μm to z=85  μm. Figures 8 and 9 (Videos 3 and 4) display the recorded images at different positions with the DH-PSF mask on and off the SLM, respectively. The reconstructed 3D image in Fig. 6 shows the eight microspheres dispersed within the onion epidermal tissue layer. The thickness of the onion epidermal layer was estimated to be 100  μm, which was in agreement with the theoretical values, and the distribution of the microspheres had a span of ∼80  μm. Once again, the image reconstructed from the DH-PSF deconvolution provides more details than that of conventional microscopy.

**Fig. 7:**
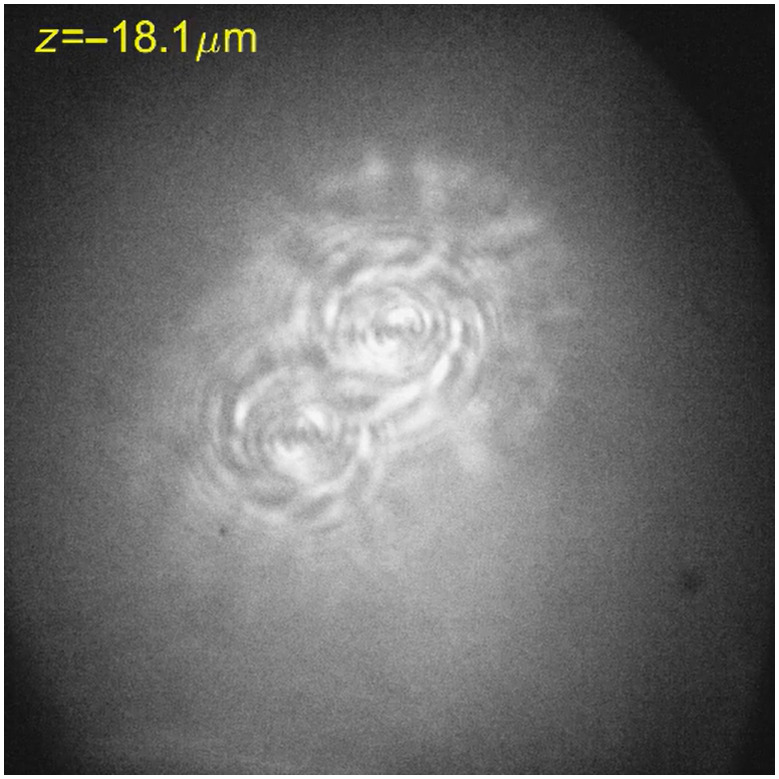
Example of recorded images at different positions with the DH-PSF mask on the SLM of sample 1 (Video 1, MP4, 14.1 MB [URL: https://doi.org/10.1117/1.JBO.28.4.046008.s1]).

**Fig. 8:**
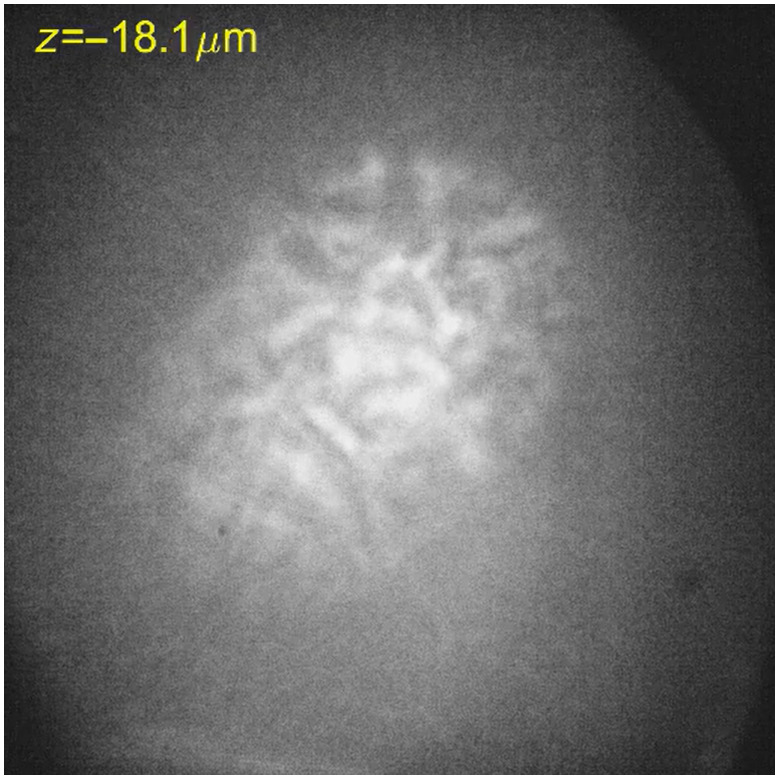
Example of recorded images at different positions without the DH-PSF mask on the SLM of sample 1 (Video 2, MP4, 14.1 MB [URL: https://doi.org/10.1117/1.JBO.28.4.046008.s2]).

**Fig. 9:**
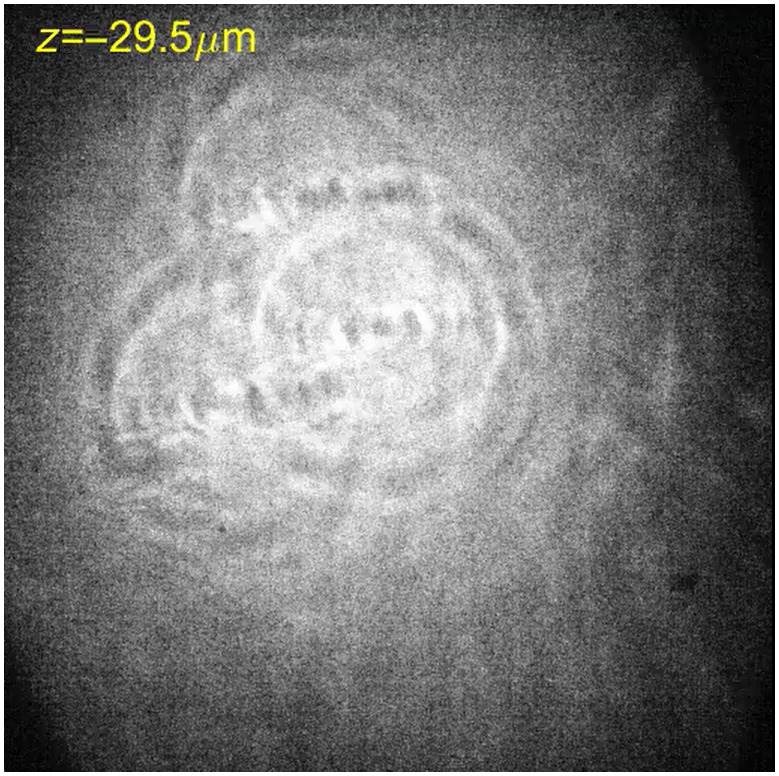
Example of recorded images at different positions with the DH-PSF mask on the SLM of sample 2 (Video 3, MP4, 11.9 MB [URL: https://doi.org/10.1117/1.JBO.28.4.046008.s3]).

**Fig. 10:**
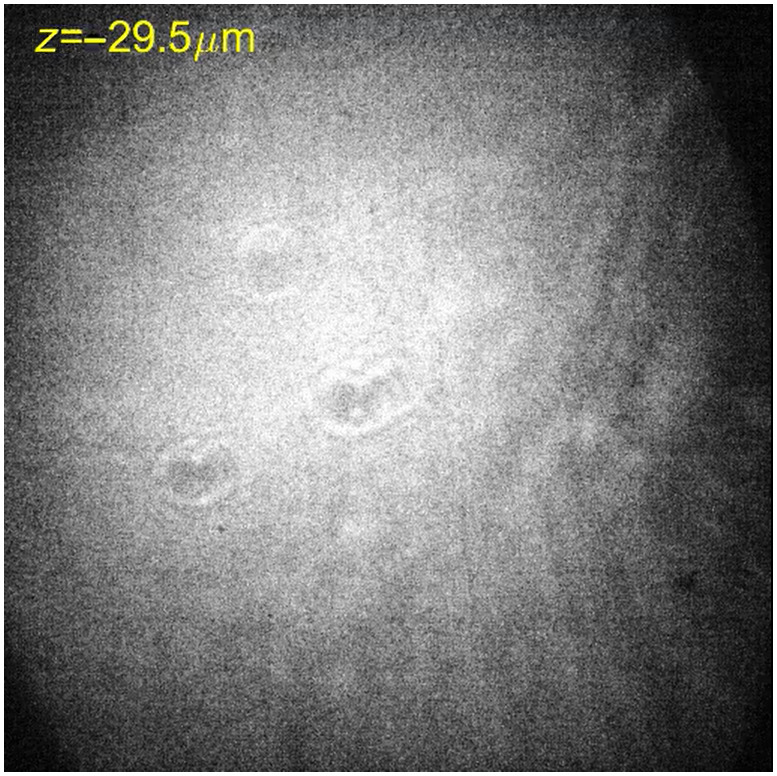
Example of recorded images at different positions without the DH-PSF mask on the SLM of sample 2 (Video 4, MP4, 11.6 MB [URL: https://doi.org/10.1117/1.JBO.28.4.046008.s4]).

## 5 Discussion and Conclusions

The DH-PSF deconvolution has an excellent optical sectioning capability, determined by the axial localization accuracy. Combined with axial scanning, we can see the internal distribution of a turbid medium and the targets of interest in/behind the medium. In this case, fluorescent staining is unnecessary as long as there is valid illumination. For sparse particles, their positions in/behind the scattering medium can be localized with the calibrated accuracy of the DH-PSF system. After staining with fluorescent dyes, sparse excitation and image fusion can achieve a super-resolution image of a target of interest. The surrounding scattering medium can also be observed with and without fluorescent staining using DH-PSF super-resolution and optical sectioning.

Currently, limited by experimental facilities in our proof-of-concept experiment, the optical sectioning and super-resolution localizing capability is demonstrated directly, whereas super-resolution imaging is reliably reasoned from localization accuracy. The implementation of DH-PSF microscopy can be realized by simply integrating a PSF modulation module into a fluorescence microscope. Next, we will image stained cells covered by scattering media to achieve super-resolution imaging of the cells and their surrounding environments. However, some issues must be addressed when using this method to image the stained cells. For instance, a high-power laser is necessary to penetrate the scattering medium and excite stained cells, which may cause photodamage or phototoxicity. This time-consuming scanning procedure restricts its application to dynamic targets and scattering media. The penetration thickness is limited because deeper tissues like the hypodermis are still unreachable. The scanning procedure may be replaced by 3D deconvolution, and the penetration thickness may be enhanced using a longer excitation wavelength or other scattering-overcoming techniques. In the future, the unprecedented super-resolution at the deep penetration of DH-PSF microscopy might enable a clear view of internal organelles and protein structures of cells in biological tissues, which is critical for investigating cell functions and metabolism *in situ* and *in vivo*.

In summary, we proposed a new application of DH-PSF microscopy. Benefitting from the optical sectioning of our developed DH-PSF deconvolution algorithm, the image of the targets of interest and the surrounding scattering medium can be significantly enhanced even without fluorescent staining. We have demonstrated that DH-FSF microscopy can be utilized for super-resolution imaging and localization through or within scattering media, and the penetration thickness increases to an OT of 5. At the same time, the transverse resolution is 13 nm, and the axial resolution is 51 nm. The ability to see and localize through or within scattering media has the potential to enable *in situ* observation of cells in tissues, which may provide a powerful tool for numerous bio-related fields.
